# Nutritional and Lifestyle Therapy for NAFLD in People with HIV

**DOI:** 10.3390/nu15081990

**Published:** 2023-04-20

**Authors:** Felice Cinque, Annalisa Cespiati, Rosa Lombardi, Giovanni Guaraldi, Giada Sebastiani

**Affiliations:** 1 Division of Gastroenterology and Hepatology, and Chronic Viral Illness Service, McGill University Health Centre, Montreal, QC H4A 3J1, Canada; felice.cinque@mail.mcgill.ca; 2 Department of Pathophysiology and Transplantation, University of Milan, 20122 Milan, Italy; 3 Medicine and Metabolic Disease Unit, Fondazione IRCCS Cà Granda Ospedale Maggiore Policlinico, Via F. Sforza 35, 20122 Milan, Italy; 4 Modena HIV Metabolic Clinic, Department of Surgical, Medical, Dental and Morphological Sciences, University of Modena and Reggio Emilia, 41125 Modena, Italy; 5 Infectious Diseases Unit, Azienda Ospedaliero-Universitaria di Modena, 41124 Modena, Italy

**Keywords:** HIV, fatty liver, MAFLD, NAFLD therapy, nutrients, lifestyle, diet, exercise, food insecurity, lean NASH, microbiota

## Abstract

HIV infection and nonalcoholic fatty liver disease (NAFLD) are two major epidemics affecting millions of people worldwide. As people with HIV (PWH) age, there is an increased prevalence of metabolic comorbidities, along with unique HIV factors, such as HIV chronic inflammation and life-long exposure to antiretroviral therapy, which leads to a high prevalence of NAFLD. An unhealthy lifestyle, with a high dietary intake of refined carbohydrates, saturated fatty acids, fructose added beverages, and processed red meat, as well as physical inactivity, are known to trigger and promote the progression of NAFLD to nonalcoholic steatohepatitis, liver fibrosis, and hepatocellular carcinoma. Furthermore, with no currently approved pharmacotherapy and a lack of clinical trials that are inclusive of HIV, nutritional and lifestyle approaches still represent the most recommended treatments for PWH with NAFLD. While sharing common features with the general population, NAFLD in PWH displays its own peculiarities that may also reflect different impacts of nutrition and exercise on its onset and treatment. Therefore, in this narrative review, we aimed to explore the role of nutrients in the development of NAFLD in PWH. In addition, we discussed the nutritional and lifestyle approaches to managing NAFLD in the setting of HIV, with insights into the role of gut microbiota and lean NAFLD.

## 1. Introduction

The global human immunodeficiency virus (HIV) epidemic is still a major health concern, with 38.4 million people having HIV (PWH) in 2021, according to the World Health Organization [1]. Tremendous scientific advances have been made, and luckily, the revolution of combined antiretroviral therapy (cART) has turned HIV infection from a fatal disease into a chronic yet treatable condition [2]. Despite its narrowing, we still witness a gap in the life expectancy between PWH and the general population [3]. Acquired immunodeficiency syndrome (AIDS) still accounts for the highest number of deaths, especially in developing countries, while the burden of non-AIDS comorbidities is increasingly growing over the time [4]. In fact, aging with HIV also leads to a higher mortality related to non-communicable diseases, mainly cancer, cardiovascular disease, and liver disease [5]. Of note is that PWH experience a higher and anticipated prevalence of noninfectious comorbidities compared to the uninfected population, making the management of non-AIDS comorbidities a priority [6]. Furthermore, liver-related diseases in PWH have risen over the years, becoming one of the most common comorbidities and among the leading causes of non-AIDS-related deaths [7]. In the setting of HIV, a variety of factors, including coinfection with hepatitis C (HCV) and hepatitis B (HBV) viruses, alcohol, the hepatotoxicity of cART, and nonalcoholic fatty liver disease (NAFLD), contribute to liver injury [8]. Notably, NAFLD is currently the leading cause of liver disease worldwide and its burden keeps increasing over time [9]. NAFLD is defined by the accumulation of fat in more than 5% of hepatocytes, detected either by imaging or histology, with no evidence of other causes of underlying liver diseases such as alcohol abuse, viral hepatitis, or genetic disorders [10]. The spectrum of this condition is wide, ranging from simple steatosis to nonalcoholic steatohepatitis (NASH), where inflammation and hepatocyte injury coexist, possibly evolving to liver fibrosis, cirrhosis, and hepatocellular carcinoma (HCC) [11]. NAFLD affects approximately 25–30% of the global population, with a much higher prevalence in high-risk groups such as patients with type 2 diabetes mellitus or obesity, where it rises to 50% and 90%, respectively [12]. Given the high prevalence of NAFLD in the context of metabolic syndrome, and considering that individuals with hepatic steatosis show a high risk of cardiovascular complications and mortality, a new definition for metabolic-dysfunction-associated fatty liver disease (MAFLD) has been proposed [13,14]. MAFLD is diagnosed by the presence of fatty liver with one of the following metabolic dysfunctions: overweight, obesity, the presence of type 2 diabetes, or a normal weight with evidence of metabolic dysregulation [14]. This term seems to better account for the strong and bidirectional association between NAFLD and metabolic syndrome [15]. Like obesity and diabetes, a growing body of evidence shows that HIV is a risk factor for hepatic steatosis. Indeed, the prevalence of NAFLD in PWH ranges from 13 to 65% [16], with two meta-analyses based on imaging studies reporting that around 35% of HIV mono-infected patients had NAFLD [17,18]. Notably, NAFLD is not only more common, but also seems to be more severe in the setting of HIV [19], with a pooled prevalence of biopsy-proven NASH and significant liver fibrosis of up to 49% and 23%, respectively [18]. Furthermore, NAFLD pathogenesis is likely more complex within PWH compared to the general population. First, classic metabolic risk factors, such as type 2 diabetes, dyslipidemia, and hypertension, are more frequent in the context of HIV [20]. Moreover, unique determinants, including HIV chronic inflammation and a lifelong exposure to cART, especially old nucleoside reverse transcriptase inhibitors (NRTIs) and ritonavir-boosted protease inhibitors (PIs), also contribute to the causative pathway towards NAFLD [16]. Among PWH, the recently introduced definition of MAFLD seems very promising, because unlike NAFLD, it does not exclude patients with alcohol abuse and an HBV or HCV coinfection, which are conditions commonly associated with HIV. In addition, the literature highlights the pivotal roles of nutrition and lifestyle in both the pathogenesis and management of NAFLD. First of all, an unhealthy diet rich in polyunsaturated fat, sugar-sweetened beverages, red and processed meat, and an excessive carbohydrates intake, along with a sedentary lifestyle and a lack of physical activity, are known risk factors for NAFLD [21]. Moreover, although this is being attempted in many randomized clinical trials (RCT), there are currently no NASH-targeted therapies that have been approved by the Food and Drug Administration (FDA), with lifestyle intervention being the cornerstone and only available treatment [10,22]. While strong evidence is available for the uninfected population, studies tailored to the HIV population are much fewer, and the roles of macronutrients and lifestyle habits in NAFLD in PWH are still unclear. Therefore, the aim of the current narrative review is to provide insight into the available literature on the roles of nutrition and lifestyle in the pathogenesis and treatment of NAFLD in PWH.

## 2. Methods

We performed a comprehensive literature search for this narrative review by screening peer-reviewed articles regarding nutrition and lifestyle in the setting of NAFLD in PWH, using the electronic database PubMed. Studies from 1996—when cART was introduced—to February 2023 and only English papers were included. The following search terms were matched to “Non-alcoholic fatty liver disease” or “Non-alcoholic steatohepatitis” and ““HIV” or “HIV infection”, or related acronyms were used: “nutrition”, “diet”, “carbohydrates”, “lipids”, “fatty acids”, “proteins”, “vitamins”, “alcohol”, “coffee”, “cannabinoids”, “food insecurity”, “lifestyle”, “exercise”, “physical activity”, “lean NAFLD”, and “gut microbiota”. We included experimental and observational studies, clinical trials, systematic reviews, meta-analyses, editorials, and commentaries reporting data about nutritional and lifestyle approaches to NAFLD in PWH. We excluded studies that did not meet the selection criteria, meeting abstracts and duplicate publications.

## 3. The Role of Nutrients in NAFLD in People with HIV

### 3.1. Nutrients in NAFLD 

#### 3.1.1. Macronutrients

Macronutrients have an impact on the multifactorial pathogenesis of NAFLD, being able to alter hepatic fat storage and liver inflammation [23]. In this section, we will discuss the roles of carbohydrates, lipids, proteins, alcohol, and coffee in NAFLD.

##### Carbohydrates

Overfeeding with refined carbohydrates leads to an intrahepatic triglycerides accumulation, supporting the role of excessive carbohydrates consumption in hepatic de novo lipogenesis [24]. Special attention should be paid to soft drinks, which are beverages made with great amounts of high fructose (corn syrup) or sucrose, having been shown to be a risk factor for NAFLD independently of metabolic syndrome [25,26]. In contrast, the consumption of whole grains, oatmeal, and quinoa, which are very rich in vitamins, minerals, and fibers, shows a strong inverse correlation with NAFLD [27,28,29] and improves insulin sensitivity, with proven benefits for metabolic disease [30,31].

##### Lipids

As for carbohydrates, the type of fat being ingested has a great impact on the NAFLD pathophysiology, more so than the total amount consumed [32]. On the one hand, the dietary abuse of saturated long-chain fatty acids (FAs), which are commonly found in animal products, promotes hepatic oxidative stress and the impairment of insulin signaling, leading to fatty liver, insulin resistance, and NASH [23]. On the other hand, plant-based food, especially nuts, olive oil, and avocados, are good sources of dietary monounsaturated FAs and polyunsaturated FAs, which are both associated with the amelioration of hepatic steatosis and liver inflammation [33,34]. In addition, among polyunsaturated FAs, there is evidence of a more harmful effect of *n*-6 FA (linoleic acid) than *n*-3 FA (alpha-linolenic acid) on the accumulation of intrahepatic triglycerides, with a higher intake of *n*-6 FAs and an increased *n*-6/*n*-3 ratio in NASH patients compared to controls [35].

##### Proteins

Finally, there is conflicting evidence regarding the association between protein consumption and NAFLD. Although a high protein intake seems to reduce intrahepatic fat, it may also negatively impact insulin sensitivity [23]. Again, the source of the protein is critical. In fact, a moderately high protein intake from white meats and legumes seems to be beneficial, lowering the hepatic fat content and markers of liver damage [36]. Conversely, eating a large amount of red and processed meat is associated with NAFLD and insulin resistance, independently of saturated fat intake [37,38].

##### Alcohol

Another macronutrient relevant to NAFLD is alcohol. By definition, the alcohol intake in NAFLD is either absent or low to mild (less than 20 g/day for women and less than 30 g/day for men) [10], but its effects on this population are still debated. Among NAFLD patients, while some old retrospective studies pointing to a favorable association between moderate alcohol use and less severe histological liver damage [39,40], more recent research has shown that even mild drinking might lead to liver fibrosis progression [41,42]. Furthermore, in a prospective cohort of NASH patients, alcohol consumption was identified as the major risk factor for HCC [43]. Evidence from longitudinal cohorts is accumulating, with a recent systematic review concluding that, in patients with NAFLD, any amount of alcohol intake, even at low concentrations, seems to be harmful to the liver’s health [44]. As for cardiovascular outcomes, limited data are available about the effects of small alcohol consumption on the cardiovascular risk in NAFLD. In fact, one study has shown a protective effect of moderate alcohol intake on cardiovascular outcomes [45], while others have found no difference [41,46]. Finally, aligned with the latest evidence, the most recent American Association for the Study of Liver Diseases (AASLD) guidance states that alcohol abstinence may lower the risk of fibrosis progression and hepatic and extrahepatic cancers in patients with NAFLD [10].

##### Coffee

Coffee is the second most consumed beverage in the world after water [47]. Coffee contains several compounds with known antioxidant effects, such as caffeine, chlorogenic acid, cafestol, kahweol, trigenolline, and tocopherols. These antioxidants are known to improve a number of liver conditions, ranging from fibrosis to cirrhosis and HCC [48]. In the setting of NAFLD, a recent meta-analysis of five cross-sectional studies showed a positive correlation between a high coffee consumption and a lower liver fibrosis, although no clear agreement was reached on the required dose [49]. However, not all forms and methods of coffee preparation show the same beneficial effects on NAFLD. Anty et al. reported that regular filtered coffee intake was associated with a lower liver fibrosis, while espresso was not [50]. In fact, antioxidant compounds seem to be better preserved in filtered coffee than in espresso [51]. In addition, espresso, as well as Turkish or Scandinavian boiled coffee, are often consumed with refined sugar, which is harmful for NAFLD, as it is highly rich in fructose [49]. Similarly, consuming a latte or cappuccino, in which the coffee is added to milk, which is rich in saturated fat, can also be detrimental [52]. Finally, in a recent RCT evaluating the administration of coffee components to treat NAFLD in diabetic patients, a supplementation with caffeine and/or chlorogenic acid did not improve hepatic steatosis or liver stiffness [53].

#### 3.1.2. Micronutrients

In addition to macronutrients, micronutrients also play a role in the complex pathogenesis of fatty liver; thus, expert opinions recommend the consumption of micronutrients with proven antioxidant and anti-inflammatory effects to prevent and treat NAFLD [54].

##### Vitamin E

Vitamin E, which similarly to polyunsaturated FAs can be found in olive oil, nuts, and green vegetables, is a lipophilic antioxidant engaged in cellular signaling and gene expression regulation, showing anti-inflammatory and anti-apoptotic properties [55]. In total, two RCTs, the PIVENS trial in adults with NASH [56] and the PIVOT trial in the pediatric population [57], showed that a vitamin E supplementation resulted in a significant resolution of NASH compared to a placebo. Although these trials were not able to demonstrate that a vitamin E supplementation reduces liver fibrosis, a recent retrospective study on 236 individuals with severe liver fibrosis and NASH found a positive correlation between vitamin E consumption and both a longer transplant-free survival and reduced rates of hepatic decompensation [58]. In line with these promising results, the administration of vitamin E is acknowledged in both the AASLD and the European Association for the Study of the Liver guidelines as a short-term therapeutic option for non-diabetic NASH patients with liver fibrosis or with a high necroinflammation and a risk of fast histologic progression [10,22].

##### Vitamin D

Vitamin D may play a role in NAFLD development, as its deficiency is able to activate Toll-like receptors, induce oxidative stress, and finally, increase liver inflammation [59]. In mouse models, low serum vitamin D promotes the progression of simple steatosis to NASH [60], while vitamin D receptor ligands prevent hepatic stellate cells activation and liver fibrosis [61]. Consistently, human studies have shown that having decreased serum vitamin D concentrations is common among NAFLD patients [62], with a recent meta-analysis reporting an inverse correlation between vitamin D serum levels and an increased risk of NAFLD [63]. While several preclinical studies have shown a beneficial role for vitamin D in fatty liver [64], so far, interventional human studies have failed to demonstrate that a vitamin D administration improves the histologic severity of NAFLD [65], although it is significantly associated with a reduced fasting glucose and insulin resistance [66]. This may be explained by the limited number of trials examining the role of vitamin D replacement in NAFLD and by the variability of the levels and methodologies used to detect serum vitamin D levels [67].

##### Polyphenols

Polyphenols, commonly found in fruits, vegetables, olive oil, coffee, red wine, and dark chocolate, might be involved in the pathophysiology of NAFLD by preventing oxidative stress and inflammation, promoting FA beta-oxidation, and modulating insulin resistance [68]. Several clinical trials have revealed intriguing early findings on the safety and efficacy of polyphenols administration for the treatment of NAFLD. Specifically, a supplementation with curcumin [69,70], Silymarin [71], or hesperidin [72] seems to improve the different features of NAFLD with a good safety profile, whereas the available evidence on resveratrol is conflicting [73]. However, despite the encouraging preliminary data, further well-designed RCTs are warranted to address the use of polyphenols in NAFLD management.

##### Cannabinoid

Another natural plant-based compound whose role is being studied in chronic liver diseases is cannabinoid. Evidence from pre-clinical models shows that a cannabinoid receptors 1 overexpression leads to de novo hepatic lipogenesis, along with an intrahepatic monounsaturated FAs accumulation. Oppositely, a cannabinoid receptors 1 blockage inhibits insulin resistance and hepatic steatosis [74]. Similarly, cannabinoid receptors 2 are known to contribute both directly—by inducing liver inflammation—and indirectly—by increasing the hepatic cannabinoid receptors 1 expression—to NAFLD, NASH, and liver fibrosis [74]. Aligned with these findings, human studies have confirmed an inverse association of cannabis consumption with NAFLD [75,76], as well as with its metabolic risk factors, namely obesity [77], diabetes [78], and metabolic syndrome [79]. However, although several preclinical studies have suggested cannabinoids as a potential treatment for NAFLD, so far, no RCTs have been able to demonstrate their effective role in reducing hepatic steatosis or fibrosis [80].

### 3.2. Nutrients in NAFLD in People with HIV

#### 3.2.1. Macronutrients

Limited data are available on the role of nutrients in the setting of NAFLD in PWH, with no studies specifically addressing the roles of carbohydrates and proteins. Studies evaluating the impact of lipids, alcohol, and coffee on hepatic steatosis and/or liver fibrosis in PWH are shown in Table 1.

##### Lipids

As for the uninfected population, fat consumption plays a role in NAFLD pathogenesis in PWH. An altered hepatic FA composition was associated with NAFLD in PWH in a Canadian study comparing 20 PWH with NAFLD to 21 NAFLD uninfected subjects and 7 healthy controls [81]. The PWH with NAFLD showed a higher hepatic *n*-6/*n*-3 ratio, with a lower *n*-3 erythrocytes polyunsaturated FAs concentration (which reflects a lower polyunsaturated FAs dietary intake), and lower levels of the indirect markers of enzymatic FA desaturation and elongation compared to the controls. Interestingly, the altered FA composition was more impaired in the PWH with NAFLD compared to the uninfected NAFLD population [81]. Based on these results, FA imbalances, which are known to be major drivers of NAFLD, seem even more prominent in NAFLD in PWH. Indeed, the association between lipid imbalances and HIV infection is well established, with a high prevalence of dyslipidemia in PWH [90]. This might be caused both by HIV chronic inflammation, since lipid alterations were already reported before the use of cART [91], and a lifelong exposure to cART, especially to old PIs boosted with ritonavir and certain NRTIs [92]. Another recent study evaluated the changes in the plasma FAs profiles between 31 HIV-positive and 22 HIV-negative subjects with NAFLD [82]. Although they didn’t report any significant differences in the overall FA compositions, the authors showed higher concentrations of α-Linolenic, trans-palmitoleic, and behenic acids, all of which have been previously associated with metabolic alterations, in the PWH compared to the uninfected population. Additionally, the PWH had a lower activity of the elongases ELOVL1 and ELOVL6 (enzymes regulating the elongation of FAs), which have previously been found to be reduced in other chronic inflammatory diseases such as psoriasis. Interestingly, despite showing a similar hepatic steatosis severity, the PWH were significantly younger, with a lower body mass index (BMI) and a lower prevalence of metabolic syndrome compared to the controls [82], suggesting that an FA imbalance might occur earlier and at a lower BMI in the context of NAFLD in PWH. Larger studies assessing the differences in the FA compositions between HIV-positive and HIV-negative NAFLD patients are required. In a well-characterized cohort of 451 HIV mono-infected patients in Brazil, De Almeida et al. showed that the odds of developing NAFLD were 91% higher in the subjects with a high usual fat consumption compared to those with a lower fat intake [83]. As expected, a moderate monounsaturated FAs ingestion was linked to lower odds of liver steatosis and fibrosis, and a moderate n-6 polyunsaturated FAs intake was associated with lower odds of NAFLD. Surprisingly, as opposed to the evidence for the general population, no association between the total saturated fat consumption and NAFLD was found. Instead, a moderate intake of saturated FA seemed to be protective against NAFLD, with lower odds of liver fibrosis compared to a lower saturated FA consumption. Moreover, unexpectedly, the odds of liver fibrosis appeared to increase with a higher *n*-6 polyunsaturated FAs intake [83]. These results might reflect the methodology of the study, which relied on the 24 h dietary recall method, which is a self-assessment tool performed only on two non-consecutive days, to define the habitual fat intake. In addition, evidence about the role of polyunsaturated FAs in the liver health of PWH is mixed. A first study comparing 216 PWH with 1604 uninfected subjects found that polyunsaturated FAs intake, *n*-6 polyunsaturated FAs, and the ratio of polyunsaturated FAs to saturated FAs were associated with liver damage, which was measured by transaminases levels [93]. Conversely, in a case-control study comparing 305 PWH to 301 healthy controls, Stonehouse and colleagues showed an inverse correlation between the plasma total omega-6 polyunsaturated FAs concentrations and liver enzymes in both HIV-infected and HIV-uninfected subjects [94]. Surprisingly, De Almeida et al. found a direct association between a higher usual intake of total carbohydrates and lower odds of NAFLD [83]. This finding is in disagreement with the large body of evidence for the uninfected population showing that high intakes of dietary sugar increase the risk of NAFLD [24]. However, although a description of the subtypes of carbohydrates ingested was not available, the authors suggested that the cohort had a high dietary intake of beans and cereals, which provide fiber and polysaccharides instead of the more harmful fructose [83]. Overall, based on the strong evidence for the general NAFLD population [10,22], PWH with NAFLD should avoid a high intake of refined carbohydrates and fructose-supplemented beverages. Finally, De Almeida and colleagues revealed that a moderate consumption of dietary fibers was associated with lower odds of NAFLD, which is in line with the evidence for the general population [27,28,29]. Focusing on omega 3 supplementation, although no study has specifically addressed NAFLD in PWH, in a meta-analysis of nine RCTs performed on PWH with hypertriglyceridemia, a diet supplementation with polyunsaturated FAs significantly improved triglycerides and high-density lipoprotein cholesterol, with a favorable safety profile [95]. Additionally, an omega-3 administration seemed to be also able to decrease systemic HIV-related inflammation, as measured by serum C-reactive protein [96]. Therefore, a dietary supplementation with omega-3 polyunsaturated FAs is recommended for PWH with hypertriglyceridemia, although further studies are warranted to evaluate its impact on NAFLD. Finally, we feel that the evidence on macronutrients for NAFLD in PWH, especially on carbohydrates and lipids, is scarce and conflicting, calling for future research.

##### Alcohol

Alcohol consumption is common among PWH and its abuse is known to be associated with unfavorable clinical outcomes [97]. In fact, in the setting of HIV, drinking alcohol may lead to a low medication adherence, as well as to poor HIV control, with decreased CD4+ cells and elevated HIV-RNA levels, regardless of cART use [98,99]. In addition, by suppressing the innate and acquired immune system and causing a disruption in the gut barrier, alcohol intake seems to increase the susceptibility to HIV infection and accelerate the disease’s progression [100,101]. As for the liver’s health, alcohol metabolism may potentiate the effects of HIV-induced hepatotoxicity, because alcohol and HIV infection act on common targets to cause liver damage [102]. Ganesan et al., in a preclinical study, demonstrated that ethanol metabolism promotes an accumulation of HIV components in hepatocytes, causing oxidative stress and apoptotic cell death [103]. The underlying mechanism is potentially related to acetaldehyde-induced lysosomes damage, which enhances this HIV-induced hepatotoxicity [104]. Of note is that the removal of these HIV-infected apoptotic bodies by nonparenchymal liver cells triggers their activation, contributing to liver disease progression [103]. Our literature search shows a lack of clinical studies evaluating the impact of light to moderate alcohol intake on the liver outcomes among PWH, especially in the setting of NAFLD. In a large prospective cohort of 686 HIV/HCV-coinfected women, with a median follow-up of 10 years, light (1–3 drinks/week) or moderate (4–7 drinks/week) alcohol consumption was not associated with liver fibrosis progression, whereas drinking more than 14 drinks per week showed increased rates of fibrosis progression [84]. Therefore, Kelly and colleagues concluded that, although heavy alcohol consumption should be strongly discouraged in this population, complete abstinence might not be necessary [84]. Interestingly, a Danish study comparing 453 PWH without viral hepatitis and an excessive alcohol consumption to 765 healthy controls, showed that, in the HIV population, a moderate alcohol consumption (defined as <14 alcoholic units per week for men and <7 alcoholic units per week for women) was associated with lower odds of moderate-to-severe hepatic steatosis, in a multivariate analysis adjusted for age, sex, ethnicity, BMI, and physical activity level [85]. In addition, although a weekly consumption of beer was associated with lower odds of moderate-to-severe hepatic steatosis, no association was found with wine, liquor, sugar-sweetened beverages, coffee, fast food, and type of meat products [85]. However, in this study, the prevalence of moderate-to-severe hepatic steatosis (assessed by computed tomography) was only 8.6%, which is very low compared to the previous evidence on NAFLD in PWH and may have led to an underestimation of the alcohol effect. Further studies assessing the role of mild alcohol intake in NAFLD in PWH are needed.

##### Coffee

**Figure 1:**
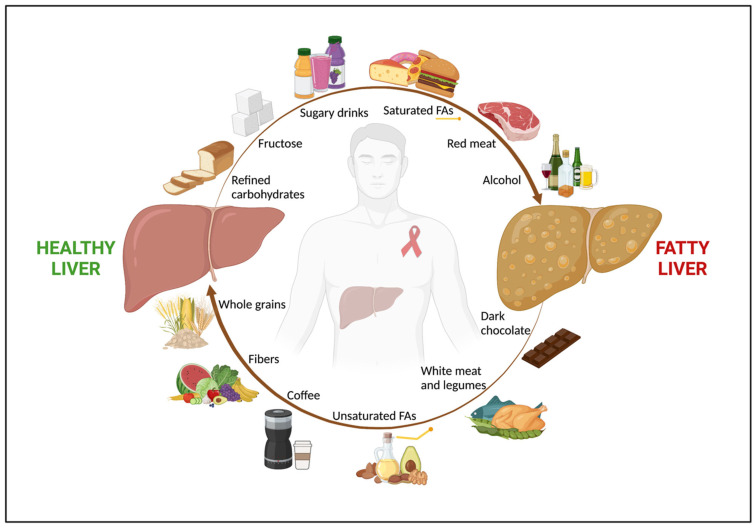
The role of nutrients in NAFLD in people with HIV. Among people with HIV, nutrition has a key impact in both NAFLD onset and regression. An unhealthy diet, rich in refined carbohydrates, fructose and sugar-added beverages, saturated FAs, and red and processed meat, along with alcohol abuse, are associated with fatty liver. Conversely, a healthy diet, with high intake of fruits, vegetables, whole grains, which are good sources of fibers, unsaturated FAs, white meat, dark chocolate, and coffee shows a beneficial effect on NAFLD. *Abbreviations*: NAFLD; non-alcoholic fatty liver disease; and FAs, fatty acids.

The anti-inflammatory and hepato-protective properties of coffee have also been studied in the setting of HIV. Evidence on this topic comes from a national multicenter prospective cohort of PWH coinfected with HCV in France (ANRS CO13 HEPAVIH) [86]. Firstly, in a subset of 990 subjects, Carrieri et al. showed that a higher coffee intake (≥three cups/day) was associated with a lower risk of impaired transaminases, with similar results for chocolate consumption [86]. Notably, combining a high intake of the two macronutrients together reduced the risk of abnormal liver enzymes by about 40% [86]. In 1028 HIV/HCV-coinfected patients with a median follow-up of 5 years, the same authors observed that a baseline high daily coffee intake of three or more cups was significantly associated with a 50% reduced risk in all-cause mortality [87]. Interestingly, looking at the baseline characteristics of the study cohort, the patients with a high coffee consumption were also less likely to present an advanced liver fibrosis in their Fibrosis-4 score (FIB-4) than the subjects with a moderate (i.e., two cups/day) or low (i.e., one cup/day, occasional consumption, or no consumption) coffee intake (10.7% vs. 14.3% vs. 19.7% respectively, *p* = 0.002) [87]. However, in this cohort, the effect of HCV infection may have been greater than that of HIV, being that HCV-related mortality was the first cause of death (42.8%), followed by non-HIV and non-HCV-related cancer (11.7%), AIDS (10.8%), and cardiovascular disease (3.9%) [87]. In a recent editorial, the same team further investigated the impact of coffee intake on significant liver steatosis and fibrosis among the patients of the ANRS CO13 HEPAVIH cohort [88]. Despite the lack of an association with hepatic steatosis, coffee intake was confirmed to have a significant protective effect on liver fibrosis, with a dose–response relationship for both the FIB-4 and liver stiffness measurements taken by transient elastography [88]. Finally, in a longitudinal observational study on 1019 subjects from the same cohort, Yaya et al. not only confirmed a strong inverse association between a high coffee consumption (i.e., ≥ three cups/day) and advanced liver fibrosis, but also showed a role for coffee in mitigating alcohol’s negative effect on liver damage [89]. Indeed, a higher coffee intake was associated with a lower risk of liver fibrosis, even in HIV/HCV-coinfected patients with a high-risk alcohol consumption (i.e., >four alcoholic units/day for men and >three alcoholic units/day for women). Although no data regarding hepatic steatosis were available, patients with a high coffee intake and low-risk alcohol consumption (i.e., ≤four alcoholic units/day for men and ≤three alcoholic units/day for women) had a significantly lower prevalence of advanced liver fibrosis (FIB-4 ≥ 3.25) compared to subjects with a low coffee intake and low-risk alcohol consumption (11.4% vs. 77.3% respectively, *p* < 0.001) [89], possibly suggesting a beneficial role of drinking coffee in NAFLD in PWH. However, since all these studies addressed a population of HIV/HCV coinfected subjects, there is still a need for evidence specifically assessing the link between coffee and NAFLD in HIV monoinfection. Figure 1 shows the role of nutrients in NAFLD in PWH.

#### 3.2.2. Micronutrients

As for the uninfected population, the roles of several micronutrients have been investigated among PWH with NAFLD. Studies addressing the role of micronutrients in hepatic steatosis and/or liver fibrosis in PWH are shown in Table 2.

##### Vitamin E

Vitamin E deficiency seems to be common among PWH. For instance, in a cohort of 107 PWH in Ghana, a high prevalence (82.5%) of serum vitamin E insufficiency was detected, with a low dietary intake of this vitamin and other antioxidant micronutrients [117]. In addition, whether high vitamin E levels reduce the progression of HIV infection to AIDS is still under debate. In a study of 311 HIV-positive men in Baltimore, patients in the highest quartile of serum vitamin E levels showed a decreased risk of developing AIDS compared to those in the lowest quartile [118]. Oppositely, Graham and colleagues reported that higher vitamin E levels pre-infection were associated with a higher mortality among 67 Kenyan women with HIV [119]. More research is required on the clinical impact of vitamin E on the immunologic features of HIV. Focusing on the liver’s health, vitamin E is recommended for NASH treatment in the general population [10,22]. One of us designed a clinical trial to specifically investigate the effect of vitamin E supplementation on NASH in PWH [105]. This Canadian open-label phase 4 single-arm trial included 27 HIV mono-infected patients with non-invasive diagnoses of NASH, defined by a controlled attenuation parameter (CAP) of at least 248 dB/m and a serum cytokeratin 18 (marker of hepatocyte apoptosis) greater than 130.5 U/l. The daily administration of vitamin E 800 IU over 24 weeks led to a decrease in alanine transaminase levels and improvements in hepatic steatosis and hepatocyte apoptosis [105]. In addition, no major side effects were registered, confirming a good safety profile [105]. However, consistent with the findings for the general NAFLD population [56,57], no improvement in liver fibrosis was reported, possibly also due to the relatively short study duration [105]. Therefore, vitamin E may serve as a “bridge therapy” while awaiting novel drugs that will comprehensively treat all the features of NASH [120].

##### Vitamin D

Vitamin D seems to also play a role in NAFLD in PWH. Importantly, serum vitamin D deficiency is common among PWH, with a higher prevalence than that in the general population [121,122]. In fact, other than the traditional risk factors, such as a lack of exposure to UVB radiation, age, and a darker skin pigmentation, HIV chronic infection and the lifelong use of cART seem to exacerbate vitamin D deficiency in the setting of HIV [122]. Screening PWH for vitamin D insufficiency is clinically relevant due to the higher prevalence of osteopenia/osteoporosis and fractures in PWH and cART-treated individuals compared to controls [123]. Focusing on liver outcomes, in the setting of HIV/HCV coinfection, several studies have shown an association between vitamin D deficiency and increased biopsy-proven liver fibrosis [106,107,108]. Similarly, in a study evaluating 86 people with HIV/HCV coinfection, Mandorfer et al. reported that a vitamin D deficiency and low CD4+ nadir were independently associated with a higher portal pressure (assessed by a hepatic venous pressure gradient) and fibrosis progression rate (computed by dividing the METAVIR fibrosis score at liver biopsy by the duration of HCV infection expressed in years) [109]. Moreover, in a cohort of 139 PWH with advanced liver disease, who were candidates for liver transplantation, Branch et al. found a very high prevalence of vitamin D deficiency (90%), which was also independently associated with cirrhosis [124]. Conversely, El-Maouche and colleagues, in a cohort of 116 HIV/HCV-coinfected patients, although vitamin D deficiency was highly prevalent, there was no reported association between low vitamin D levels and liver fibrosis severity (by liver biopsy) or low bone mineral density (by dual-energy X-ray absorptiometry) [110]. Of note is that the study’s participants were predominantly African-American (87%) [110], raising questions about the role of ethnicity in the association between vitamin D deficiency and liver damage. As for NAFLD in PWH, in a cross-sectional study of 707 PWH without alcohol abuse and viral hepatitis, Milic et al. found an association between low serum vitamin D levels and liver fibrosis among the NAFLD subjects (odds ratio 1.94, 95% CI 1.18–3.24) [111]. Finally, in a cross-sectional study of 94 HIV patients, Moreno-Perez and colleagues found that vitamin D insufficiency was associated with an impaired insulin sensitivity [125], which has a well-known pathogenetic role in NAFLD [126]. Future RCTs are warranted to better clarify the role of vitamin D in NAFLD pathogenesis among PWH. These studies would also address the need to screen for vitamin D deficiency, as well as to detect and possibly manage osteoporosis in the setting of NAFLD in PWH.

##### Cannabinoids

In HIV infection, preliminary evidence has shown that cannabinoid intake may have a beneficial impact on its clinical outcomes. In fact, cannabis usage seems to be associated with lower levels of T-cell activation, inflammatory monocytes, and pro-inflammatory cytokine secretion, all of which correlate with both HIV progression and its comorbidities [127]. A few studies have investigated the role of cannabinoids in the liver’s health among PWH, with mixed results. The investigators of the ANRS CO13 HEPAVIH cohort demonstrated that, in the setting of HIV/HCV coinfection, daily cannabis use was negatively associated with being overweight [128], insulin resistance [129], and fatty liver, diagnosed by abdominal ultrasound [112] or the fatty liver index [113]. Oppositely, in 248 Russian PWH with heavy alcohol consumption, who were mostly male (72.6%), young (median age of 33.9 years), and coinfected with HCV (87.9%), Fuster et al. did not report any association between cannabis use and advanced liver fibrosis (detected by FIB-4, aminotransferase-to-platelet ratio (APRI), or transient elastography) [114]. Along the same lines, in a large longitudinal cohort of 575 HIV/HCV-coinfected women who were followed for a median of 11 years, marijuana usage was not associated with a progression to significant liver fibrosis (diagnosed by FIB-4 or APRI) [115]. Similar results were also shown by Brunet and colleagues, who detected no evidence that marijuana smoking accelerated significant liver fibrosis progression (detected by APRI) or cirrhosis in a prospective cohort of 690 Canadian HIV/HCV-coinfected individuals [116]. However, a longer follow-up than that of this study (2.7 years, interquartile range 0.8–3.8) may be needed to detect a significant rate of liver fibrosis and cirrhosis progression [116]. Of note is that recreational and medical cannabis use is also associated with negative cardiovascular, respiratory, cognitive, and psychological effects, although a definitive causal relationship between cannabis use and these adverse effects is lacking [130]. Therefore, these risks should be carefully considered before suggesting the use of cannabinoids. In addition, despite preclinical studies that have focused on isolated phytocannabinoids as therapeutic options for NAFLD showing promising results, preliminary human trials have failed to confirm them [74]. We believe that further evidence on safety and tolerability is needed before conducting large-scale efficacy human trials. In fact, as recently reported by one of us in a randomized interventional pilot study investigating the safety and tolerability of oral cannabinoids in 10 PWH, 2 patients developed elevated transaminases when receiving high doses of cannabinoids (800 mg per day) [131]. Therefore, it is still unclear what the optimal dose of cannabinoids is to determine their anti-inflammatory properties without causing hepatotoxicity.

### 3.3. Food Insecurity

When discussing nutrition and chronic diseases, in addition to the type and amount of nutrients consumed, it is relevant to mention food insecurity. Food insecurity is defined as the inability to acquire or consume an adequate diet quality or a sufficient quantity of food in socially acceptable ways, or the uncertainty that one will be able to do so [132]. Notably, it is not only a concern for low-income countries, but also for high-income ones, as it affects more than 12% of households in the United States [133]. As shown in Figure S1 (Supplementary Materials), a growing body of evidence shows an association between food insecurity and cardio-metabolic risk [134]. In fact, facing food shortages leads people to engage in compensatory behaviors, such as a lower fruit and vegetable intake, skipping meals, and cycling food restrictions, along with a higher consumption of inexpensive junk foods with poor nutritional values [135]. Indeed, all these poverty-related unhealthy habits can lead to an increase in metabolic syndrome, obesity, hypertension, and diabetes [136]. The liver’s health is also dramatically affected by health disparities, with social determinants being crucial to the development and management of chronic liver diseases [137]. Focusing on hepatic steatosis, Golovaty et al., in a large cohort of 2627 adults in the United States, reported that food-insecure subjects were more likely to have NAFLD (assessed by the fatty liver index) and advanced liver fibrosis (assessed by NAFLD fibrosis score) compared to food-secure adults [138]. Of note is that in another, even larger population-based study, including 4816 subjects with NAFLD (diagnosed by fatty liver index) and 1654 patients with advanced liver fibrosis (diagnosed by NAFLD fibrosis score, APRI, or FIB-4), food insecurity was an independent predictor of a higher mortality rate for both groups, also being significantly associated with a greater outpatient health care utilization among the NAFLD subjects [139]. In addition, PWH seem to be even more vulnerable to food insecurity than the general population, being that HIV infection is disproportionately affected by economic disadvantages and social stigmas [140]. Consistent with the findings for the general population, Muhammad et al. reported that food-insecure PWH had a poor diet quality, with a low dietary intake of fiber, vitamin E, folate, magnesium, and copper [141]. Among 603 subjects of the Miami Adult Studies on HIV cohort (43.6% HIV-positive), who were screened for NAFLD and liver fibrosis with magnetic resonance imaging, Tamargo and colleagues showed that food insecurity was linked to increased odds for NAFLD at a higher BMI, and that it was independently associated with a higher risk of liver fibrosis [142]. Finally, we would like to emphasize that the health promotion of PWH with NAFLD cannot be limited to nutritional advice alone. In fact, it is a matter of human rights to ensure that all patients, even the most underprivileged and those with the lowest incomes, have equal access to quality food. It is critical that health policies take action to remove the socioeconomic barriers that force these vulnerable patients into food insecurity, exposing them to an increased risk of serious illness and death.

## 4. Nutrition, Lifestyle and Pharmacotherapy in the Treatment of NAFLD in People with HIV

### 4.1. Lifestyle Interventions in NAFLD in People with HIV

#### 4.1.1. Lifestyle Treatment of NAFLD

Lifestyle interventions are structured programs that help patients to change their nutritional habits and exercise routines, and to learn behavioral strategies aimed at improving a range of health outcomes [143]. Current international guidelines state that, in overweight or obese NAFLD patients, the primary goal of lifestyle intervention is a 7–10% reduction in their body weight [10,22]. In fact, it is well-proven that losing weight has favorable effects on hepatic steatosis, liver inflammation, and fibrosis [144]. Weight loss can be achieved through diet, physical activity, or, even better, a combination of them [145]. Whether or not there is an ideal diet for NAFLD is still a debated issue [146]. Certainly, the Mediterranean diet has been the most widely studied in the context of NAFLD, with consistent effects on the improvement of liver disease [147,148] and a known beneficial impact on cardiovascular disease and diabetes prevention [149]. Recent clinical studies comparing the Mediterranean diet with other nutritional strategies in the setting of NAFLD have shown comparable positive effects for different diets on hepatic and metabolic outcomes [150,151]. Similarly, a low free sugar diet, characterized by the avoidance of foods with added monosaccharides and disaccharides, has emerged as a successful strategy for improving hepatic steatosis and fibrosis, as well as glycemic control and inflammation, in NAFLD patients [152]. Therefore, the latest AASLD guidance recommends a patient-tailored nutritional approach, stating that different calorie restriction strategies (a Mediterranean diet, low-carbohydrate and low-fat diets, saturated vs. unsaturated fat diets, and intermittent fasting) have a similar ability to improve NAFLD [10]. As for physical activity, international hepatologic societies, aligned with the guidelines for cardiovascular disease prevention [153], recommend that NAFLD patients engage in at least 150 min of exercise per week, with possibly a minimum of three–five training sessions [10,22]. Indeed, exercise shows a dose–effect relationship with NAFLD remission, with vigorous physical activity showing the most valuable impact [154]. However, any level of physical activity is proven to be useful, and even a small amount of exercise is better than continuing with inactivity [155]. Aerobic workouts are generally preferred, although resistance training has shown equally positive outcomes, representing a viable option for NAFLD treatment [156,157,158]. Therefore, patients should be provided with an individually tailored exercise plan, advising them to choose the activity that suits them the most. This seems to be the best strategy for improving long-term adherence, which is essential to avoiding losing the benefits that have been achieved [159]. In addition, another very effective therapy for losing weight and maintaining it in morbid obese patients is bariatric surgery, including sleeve gastrectomy and a Roux-en-Y gastric bypass, which act by reducing the volume of food that can be ingested and causing malabsorption, or a combination of these [160]. Notably, lifestyle treatment of NAFLD is associated with a reduction in liver fat and other metabolic benefits independently of weight loss [161,162,163]. In fact, following a healthy diet and engaging in physical activity promotes biological changes such as modifications in energy balance, circulating lipids, and insulin sensitivity, which can lead to NAFLD remission even without losing weight [164]. Although highly relevant, lifestyle intervention should be prescribed more cautiously in liver cirrhosis than in the earlier stages of NAFLD. As for diet, cirrhotic patients should receive nutritional counseling tailored to their current nutritional status, following a comprehensive screening for malnutrition, sarcopenia, and frailty [165]. Weight loss should be prescribed with extreme caution in overweight/obese cirrhotic patients, especially if they have decompensated cirrhosis or sarcopenic obesity [166], possibly through a multidisciplinary approach to ensure an adequate protein intake (1.2–1.5 g/kg/day). Similarly, despite exercise having been shown to improve cardiopulmonary performance, muscle mass, contractile function, and the quality of life in cirrhotic patients, it should be carefully monitored by experienced physical therapists after a precise assessment of the patient’s needs [165].

#### 4.1.2. Lifestyle Treatment of NAFLD in People with HIV

**Figure 2:**
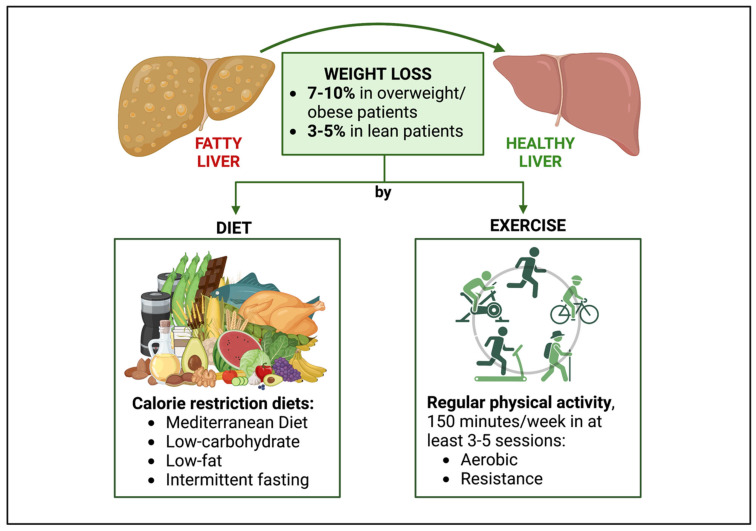
**Lifestyle treatment of NAFLD in people with HIV.** The goal of lifestyle treatment for NAFLD in PWH is weight loss, with a target of 7–10% in overweight/obese patients and 3–5% in lean individuals. Weight loss can be achieved through a patient-tailored approach, consisting of diet, physical activity, or, preferably, a combination of them. Calorie restriction diets such as Mediterranean diet, low-carbohydrate diets, low-fat diets, and intermittent fasting are recommended. Regarding physical activity, patients are advised to engage in at least 150 min of exercise (aerobic or resistance) per week, with possibly a minimum of 3–5 training sessions. *Abbreviations*: NAFLD, non-alcoholic fatty liver disease.

Figure 2 summarizes the lifestyle treatment of NAFLD in PWH. Providing good lifestyle counselling to PWH with NAFLD can be challenging. As already mentioned, PWH seem to have a poorer nutritional quality, greater rates of food insecurity, and a reduced likelihood of fulfilling their global physical activity recommendations compared to the uninfected population [167,168,169]. Furthermore, in the context of HIV, more intense physical activity may be required to achieve similar metabolic improvements to those of HIV-negative subjects [170]. Indeed, weight loss strategies of a proven efficacy for the uninfected population might not be generalizable to PWH, in whom ectopic adipose tissue, dysfunctional subcutaneous fat, and immune activation play a key role [171]. However, this should not discourage physicians, who are recommended to provide PWH with lifestyle intervention, since several studies showed that structured behavioral programs lead to weight loss and a reduction in abdominal obesity among obese PWH [172,173,174,175,176,177]. Focusing on NAFLD in PWH, evidence from the few pharmacological RTCs available reports that PWH struggle to achieve a healthy lifestyle, even when they receive comprehensive counselling [105,178]. For instance, in a trial evaluating the vitamin E effect on NASH in PWH, lifestyle changes, provided by a dedicated counseling approach, were ineffective, with no BMI improvement observed in the 21 overweight patients of the cohort after 48 months of follow-up [105]. Similarly, in the study evaluating the effects of tesamorelin on NAFLD in HIV, although all the patients were given nutritional counselling from nutritionists at baseline, 6 months, and 12 months, they did not significantly lose either weight or waist circumference by the end of the trial, with no change in their daily caloric, macronutrient, alcohol intakes or hours of physical activity [178]. It should be noted that these studies were not designed to assess the impact of lifestyle interventions, so it is possible that the participants relied on the novel drug as a cure for NAFLD, while regarding the behavioral advice as less important. Nevertheless, a lack of lifestyle changes may also lie in the peculiarities of the HIV population, such as their socio-economic vulnerability, with a low access to quality food and structured training sessions, and in the lipodystrophy and HIV-related stigma that may prevent their participation in traditional weight management programs [120,179]. A recent study tailored to NAFLD in PWH showed the benefits of a structured lifestyle intervention on weight loss [180]. In this Portuguese trial, 55 PWH with ultrasound-proven NAFLD were allocated to dietary interventions based on the Mediterranean diet (intervention group, 27 patients) or to the standard of care (control group, 28 patients), and after a 5-month follow-up, the standard of care group had gained a median of 0.65 kg, while the intervention group had lost a median of 1.5 kg (*p* < 0.001) [180]. Right after that, the 3-month period of the Coronavirus disease 2019 (COVID-19) national lockdown started, and both groups started to gain weight [180]. In fact, the COVID-19 lockdown posed a threat to healthy lifestyles, as proven by several studies reporting weight gains and the worsening of the disease control in the overall NAFLD population [181,182,183]. However, patients in the intervention group experienced less weight gain (3.1 ± 1.6 kg vs. 0.7 ± 1.7 kg, *p* < 0.001) and lower alterations in their dietary habit patterns compared to the controls, also being more likely to maintain their physical activity levels [180]. This study suggests that a structured nutritional intervention can mitigate the negative impact of lockdown on NAFLD in PWH. Another interesting aspect of this paper was the use of telemedicine to provide dietary interventions, with follow-up consultations performed by video and/or phone during lockdown [180]. Studies addressing the role of telemedicine in the NAFLD-uninfected population are accumulating, emphasizing the beneficial effects of e-Health in reducing body weight, BMI, and transaminases levels, as it increases patients’ adherence to diet and physical activity recommendations [184]. Similarly, in the setting of NAFLD in PWH, digital health seems to be a promising strategy for delivering lifestyle interventions [180], although further studies are needed. Focusing on the role of physical activity, Yarasheski and colleagues randomly assigned 44 PWH with a baseline insulin resistance and central adiposity to either pioglitazone (30 mg/day) alone or pioglitazone with supervised, progressive aerobic, and resistance exercise training (1.5–2 h/day, 3 days/week) [185]. Combining pioglitazone with a physical activity intervention resulted in further improvements in peripheral insulin sensitivity compared to pioglitazone administration alone [185]. Notably, although it did not reach statistical significance, pioglitazone plus exercise reduced the liver lipid content (measured by liver proton magnetic resonance spectroscopy) more than pioglitazone alone (−2.6 ± 1.4% vs. −1.5 ± 1.3, *p* = 0.6), hinting at a potential beneficial role of exercise training in NAFLD in PWH [185]. Table 3 summarizes the studies evaluating the role of lifestyle intervention in weight loss in hepatic steatosis in PWH. Future RCTs are warranted to specifically address the impact of a structured and tailored lifestyle intervention on PWH with NAFLD.

### 4.2. The State of the Art NAFLD Pharmacotherapy in People with HIV

A few pilot studies have assessed NAFLD pharmacotherapy in PWH (Table 4). We have already discussed the role of vitamin E supplementation as a NASH treatment in the setting of HIV [105]. A total of two trials have assessed the role of pioglitazone, a thiazolidinedione agent able to improve insulin sensitivity, in the management of NAFLD in PWH [186,187]. In a 48-week double-blind RCT, the administration of pioglitazone (45 mg/day) to 13 HIV/HCV-coinfected patients with hepatic steatosis led to a significant reduction in their intrahepatic fat content, which was assessed by magnetic resonance spectroscopy, in comparison to those who received a placebo, with no improvement in biopsy-proven NASH [186]. In a second trial, 98 HIV-positive individuals with prediabetes, who had evidence of fatty liver assessed by abdominal ultrasound or CAP ≥ 238 dB/m, were randomly assigned to pioglitazone (30 mg/day) or a placebo and followed for 48 weeks [187]. The pioglitazone administration led to a significant reduction in the CAP and liver stiffness measured by transient elastography [187]. However, in the pioglitazone group, although they were statistically significant, the mean changes in both the CAP and liver stiffness were small (−23.5 dB/m and −0.184 kPa, respectively), raising doubts about the clinical relevance of these results. In addition, no information about the changes in the prevalence of clinically significant or advanced fibrosis were provided by the authors [187]. The ARRIVE trial evaluated the effect of Aramchol (600 mg/day) an oral stearoyl-coenzyme-A-desaturase-1, an inhibitor known to reduce hepatic steatosis in the uninfected NAFLD population, among 25 PWH with NAFLD, defined by magnetic resonance imaging-proton density fat fraction vs. a placebo [188]. During a follow-up of 12 weeks, Aramchol did not reduce either hepatic steatosis nor the liver stiffness measured by magnetic resonance elastography or transient elastography [188]. Similarly, it did not change their body fat and muscle compositions, as assessed by magnetic resonance imaging and bone densitometry [188]. Another trial evaluated the therapeutic effect of tesamorelin (2 mg/day), a growth-hormone-releasing hormone analog approved by the FDA for HIV-associated lipodystrophy, among 61 PWH with NAFLD diagnosed via magnetic resonance spectroscopy [178]. After 12 months of follow-up, tesamorelin reduced the hepatic fat content by 37% and showed a lower rate of biopsy-proven liver fibrosis progression in the interventional group compared to the placebo group (10.5% vs. 37.5%, *p* = 0.04) [178]. However, no changes in the histologic NAFLD activity scores, lobular inflammation, or hepatocellular ballooning were observed [178]. Finally, in a maraviroc add-on for steatohepatitis in HIV-infected patients study, 13 PWH with biopsy-proven NASH were invited to add maraviroc, an antiretroviral agent with promising effects on NAFLD remission, to their existing suppressive cART regimen [189]. In this trial, after 48 weeks of follow-up, the liver biopsies did not show any changes in the hepatic steatosis, liver fibrosis, or NASH features in the study cohort [189]. Unfortunately, the number of trials addressing NAFLD pharmacotherapy for PWH is much fewer than that of the RCTs for the HIV-uninfected NAFLD population. In fact, any novel drug trial for NASH still considers HIV infection to be an exclusion criterion, raising the ethical issue of preventing a vulnerable group from receiving potentially beneficial treatments. Although regulatory agencies justify this decision by the concern of interactions between cART and the experimental drug, experts in the field of NASH in PWH advocate for the design of more inclusive trials [190]. A good strategy would be to incorporate a priori subanalyses to investigate HIV-unique features, especially drug–drug interactions, while keeping the same therapeutic targets, as this will prevent a protracted delay in the release of new medications for PWH [191]. For all these reasons, as is the case for the uninfected population, lifestyle intervention remains as the cornerstone treatment for NAFLD in PWH [192].

### 4.3. Lifestyle Treatment of Lean NAFLD in People with HIV

Lean NAFLD, defined as NAFLD with a BMI of <25 Kg/m^2^ among non-Asian subjects and <23 Kg/m^2^ among Asian ones, is a relevant condition in the setting of HIV. In fact, in a study from one of the authors, up to 35% of PWH with NAFLD were lean, and 24% of HIV patients presented with hepatic steatosis [193]. An even higher prevalence of lean NAFLD (45%) was reported in a cohort of Indian PWH [194]. These rates are much higher compared to the general NAFLD population, in which 7 to 20% of individuals have a normal body weight [195]. Indeed, PWH with NAFLD may be leaner than HIV-negative ones, with a case-control study that compared 26 PWH and 25 HIV-uninfected people with biopsy-proven NAFLD reporting that the PWH had a lower BMI, lower percentage of fat mass, and higher physical activity level compared to the controls [196]. Therefore, when discussing lifestyle treatment for NAFLD in PWH, the specifics of the lean population should be addressed. The recent American Gastroenterological Association guidelines recommend a modest weight loss of 3–5% in lean subjects with NAFLD, achieved through diet, exercise, and an avoidance of fructose-added drinks [195]. In fact, a lower weight loss is necessary for lean patients in the treatment of NAFLD, with an RCT showing that non-obese patients achieved NAFLD remission with a weight reduction of 3–5%, while obese participants needed at least 7–10% [197]. The pharmacotherapy of lean NASH is still an unknown field. Not only are there no specific pharmacological RTCs addressing this population, but also most of the existing NASH trials exclude non-overweight patients by protocol. Since PWH with NAFLD are denied the opportunity to be enrolled in drug RCTs, unsurprisingly, our literature search showed a lack of studies assessing the therapy for NAFLD in normal-weight PWH, which deserves future investigations.

### 4.4. The Role of Gut Microbiota in Lifestyle Treatment of NAFLD in People with HIV

Understanding the relationship between intestinal microbiota and the nutritional approaches to NAFLD in PWH is critical. The gut microbiota are an ecosystem of bacteria that coexist in dynamic balance with the host and each other, and are involved in a range of interactions that influence the host’s health across their lifespan [198]. The main contributors to the intestinal flora of healthy adults are four phyla, namely Firmicutes, Bacteroidetes, Proteobacteria, and Actinobacteria [199]. Recently, gut microbiota have been shown to play a key role in metabolic diseases, with a strong association between intestinal dysbiosis and obesity [200,201], metabolic syndrome [202], diabetes [203], and cardiovascular disease [204]. As the hepatic manifestation of metabolic syndrome, NAFLD is also strictly linked to gut microbiota disruption, with both animal and human studies clearly showing its correlation with NAFLD onset and progression [205,206,207]. Furthermore, intestinal flora play a central role in the nutritional therapy for NAFLD, as they influence the energy uptake, nutrient absorption, and fat storage from the food consumed [208]. In fact, gut bacteria profiles have been shown to differ between normal-weight and overweight individuals, with an obese microbiome harvesting more energy from the food eaten compared to a lean microbiome [200]. In more detail, weight gain and metabolic syndrome are associated with a low richness in the intestinal microorganisms [209], and the same has also been reported among NAFLD patients [210]. In addition, an obese dysbiosis is characterized by higher levels of Firmicutes and lower proportions of Bacteroidetes [201,211]. Similarly, many efforts are being made to identify a specific NAFLD microbiome signature, still with mixed results. Loomba et al. built a metagenomic-based model to detect advanced liver fibrosis among NASH patients, based on increased Proteobacteria levels and a decrease in Firmicutes [212]. This approach is promising, because identifying unique microbiome signatures for NAFLD, NAFLD fibrosis, and cirrhosis could serve as future noninvasive diagnostic or prognostic biomarkers [213]. Targeting the microbiota might also be an effective strategy for NAFLD treatment, as it could lead to the recovery of intestinal inflammation and restoration of gut permeability. Although preliminary data show that antidiabetic medications, such as biguanides/metformin, dipeptidyl peptidase 4 inhibitors, glucagon-like peptide 1 receptor agonists, and sodium-glucose cotransporter-2 inhibitors, may achieve metabolic improvements by healing microbiota dysbiosis, no drugs are currently approved [214]. Therefore, once again, a nutritional approach is recommended, as dietary fiber intake has been shown to significantly change the gut microbiota by decreasing the pathogenic bacteria and increasing the bifidobacteria, with a beneficial metabolite production and an improved barrier permeability [215]. Notably, HIV infection itself is linked to intestinal flora alterations [216,217]. Early after infection, PWH develop gut dysbiosis, characterized by a reduced diversity within the microbiome, similar to the findings for obese and diabetic patients [218]. Microbiota disruption persists in chronic HIV infection, and unexpectedly, it is not only reported in untreated subjects [219], but also in patients undergoing cART [220]. Of note is that cART initiation could further reduce the diversity of intestinal flora [219,221]. Interestingly, the similarities in the gut dysbiosis between PWH and obese or diabetic subjects may partly explain the high prevalence of metabolic comorbidities, as well as NAFLD, in the setting of HIV. Another clue about the close relationships between HIV, metabolic diseases, and the microbiome lies in the pharmacodynamics of maraviroc. Maraviroc is a CCR5 antagonist currently approved for HIV treatment that also shows anti-inflammatory properties and beneficial effects on glucose and lipid metabolism [222]. In a preclinical study, Pérez-Matute and colleagues demonstrated that a maraviroc treatment significantly changed the microbiomes of mice by reducing Enterobacteriales, a bacterial order that, in the same cohort, was positively associated with weight gain, insulin resistance, and fatty liver [223]. Under these assumptions, maraviroc could also be a valuable treatment for NAFLD in PWH, although a clinical trial failed to demonstrate its efficacy [189]. Focusing on the role of microbiota in the nutritional approaches to NAFLD in PWH, Yanavich et al. revealed a distinct microbiome profile for liver steatosis and fibrosis among 82 HIV monoinfected patients divided into a steatosis group, fibrosis group, and normal group, according to transient elastography results [224]. Some microbial perturbations were previously identified in other cohorts of NAFLD-uninfected patients, including increases in *P. copri*, while others, such as *E. rectale*, *F. magna*, *F. prausnitzii*, *A. muciniphila*, *B. fragilis*, *B. dorei,* and various species of Prevotella, were specific to this study population [224]. Surprisingly, the authors reported no significant differences in the macronutrient intakes between the normal, steatosis, and fibrosis groups, suggesting a lack of association between diet and gut dysbiosis [224]. This result is in contrast with the large body of evidence for the uninfected population, which identifies diet as a strong modulator of the microbiome composition [225,226,227,228]. In another study of 33 biopsy-proven PWH with NAFLD, Maurice et al. did not find any association between NAFLD or liver fibrosis and a distinct gut microbial signature, although they reported significant differences in the intestinal flora compositions between the PWH and an age- and sex-matched healthy control group [229]. This might be ascribed to methodological limitations, such as the small sample size or the use of markers of bacterial translocation (lipopolysaccharide-binding protein, bacterial DNA, and lipopolysaccharide), lacking robust and reproducible assays. Nevertheless, discordances among the studies examining the role of the gut microbiome in liver diseases are significant, and associations of specific bacterial populations with NAFLD are rarely replicated in subsequent research. This may be due to the extreme heterogeneity of microbiome sequencing methods, liver disease diagnostic techniques, and disease severity, as well as the clinical and demographic features among the different studies. We feel that further investigations are required to better elucidate the role of gut microbiota in PWH with NAFLD, together with the impact of dietary pattern changes on the intestinal dysbiosis of PWH.

## 5. Conclusions

Our review shows a lack of studies specifically addressing nutritional and lifestyle therapies for NAFLD in the setting of HIV. A fatty acids imbalance seems to be associated with hepatic steatosis in PWH and, in line with evidence for the uninfected population, PWH with NAFLD should avoid a diet that is rich in saturated fatty acids, refined carbohydrates, fructose-added beverages, and red processed meat. Although strict alcohol abstinence is recommended for patients with liver cirrhosis [165,230], future research on PWH with NAFLD will investigate whether a light alcohol consumption should also be completely avoided. Conversely, black coffee seems to have a beneficial impact on liver fibrosis in HIV/HCV coinfection, with a need for confirmation in HIV monoinfection. Moreover, vitamin E administration has a proven efficacy for NASH in PWH, while the roles of vitamin D supplementation and oral cannabinoids need to be further explored. Indeed, with no current approved medication and novel pharmacological NASH trials still excluding PWH, diet and exercise remain the cornerstones of NAFLD treatment in PWH, with weight loss targets of 7–10% and 3–5% in overweight/obese and lean individuals, respectively. Importantly, any successful nutritional intervention should also address food insecurity, which has been shown to worsen liver and cardiovascular diseases in PWH. Future research should focus on tailoring lifestyle therapy for NALFD to the specific needs of PWH, possibly using telemedicine and the engagement of PWH to increase patients’ awareness and adherence.

## Figures and Tables

**Table 1:** Studies evaluating the role of macronutrients in hepatic steatosis and/or liver fibrosis in people with HIV.

Author, Year, Country	Study Design	Nutrients	Study Cohort	Patients’ Characteristics (Sex, Age, BMI)	HIV Features (Duration of HIV Infection, cART, Viral Suppression)	Diagnostic Method for NAFLD/ NASH/Liver Fibrosis	Main Findings
Arendt et al., 2011, Canada [81]	Cross-sectional	Fatty Acids	48 participants: - 20 HIV-positive patients with NAFLD (*HIV/NAFLD*) - 21 HIV-negative patients with NAFLD (*NAFLD*) - 7 healthy controls	- *HIV/NAFLD*: M 100%, age 44.5 (31–70) years, BMI 25.7 (20.8–31.6) kg/m^2^ - *NAFLD*: M 100%, age 41 (18–69) years, BMI 29.1 (21.3–41) - *Controls*: M 100%, age 47 (40–64) years, BMI 28.2 (25–30.5) kg/m^2^	*HIV/NAFLD*: 95% on cART,	HS: liver biopsy	- *HIV/NAFLD* had altered hepatic FAs composition, with higher *n*-6 polyunsaturated FAs and *n*-6/*n*-3 ratio vs. *NAFLD* and controls - *HIV/NAFLD* had lower dietary intake of *n*-3 polyunsaturated FAs vs. controls - *HIV/NAFLD* had lower indirect markers of enzymatic FAs desaturation and elongation vs. controls
Martínez-Sanz et al., 2022, Spain [82]	Cross-sectional	Fatty Acids	53 participants: - 31 HIV-positive patients with NAFLD (*HIV/NAFLD*) - 22 HIV-negative patients with NAFLD (*NAFLD*)	- *HIV/NAFLD*: M 87%, age 56 (46–61) years, BMI 27 (25–28) kg/m^2^ - *NAFLD*: M 40%, age 60 (58–71) years, BMI 33 (31–36) kg/m^2^	*HIV/NAFLD*: 100% on cART, 100% with viral suppression	HS: Abdominal Ultrasound	- No difference in overall FAs composition between *HIV/NAFLD* and *NAFLD*. - *HIV/NAFLD* had higher concentrations of α-Linolenic, trans-palmitoleic, and behenic acids vs. *NAFLD* - *HIV/NAFLD* had lower activity of the elongases ELOVL1 and ELOVL6 vs. *NAFLD*
De Almeida et al., 2021, Brazil [83]	Cross-sectional	Fatty Acids, Carbohydrates, Fibers	451 PWH	M 40%, age 45 (IQR 36–53) years, BMI 25 (23–29) kg/m^2^	Duration HIV 10 (5–17) years, 97% on cART, 84% with viral suppression	- HS: CAP - Liver fibrosis: LSM by TE	- High FAs intake was associated with 91% higher odds of NAFLD - Moderate monounsaturated FAs intake was associated with lower odds of NAFLD and liver fibrosis - Moderate intake of saturated FAs was associated with lower odds of liver fibrosis - Moderate n-6 polyunsaturated FAs intake was associated with lower odds of NAFLD but higher odds of liver fibrosis - High total carbohydrates intake was associated with lower odds of NAFLD - Moderate fiber intake was associated with lower odds of NAFLD
Kelly et al., 2017, Canada/U.S. [84]	Prospective, median FU 10 years	Alcohol	686 HIV/HCV-coinfected patients	F 100%, age 39.7 (±6) years, BMI 26.1 (±6), kg/m^2^	29% on cART	Liver fibrosis: FIB-4	- Light (1–3 drinks/week) or moderate (4–7 drinks/week) alcohol intake was not associated with liver fibrosis progression - High alcohol intake (>14 drinks/week) was associated with increased rates of fibrosis progression
Kirkegaard-Klitbo et al., 2020, Danmark [85]	Cross-sectional	Alcohol	453 PWH	M 86%, age 52.4 (46.8–61.0) years, BMI 24.7 (22.4–27.5) kg/m^2^	Duration HIV 16 (8.3–23.1) years, 99% on cART, 97% with viral suppression	HS: computed tomography	- Moderate alcohol intake (<14 alcoholic units/week for men and <7 alcoholic units/week for women) and beer consumption were associated with lower odds of moderate-to-severe HS - No association between wine, liquor, sugar-sweetened beverages, coffee, fast food, type of meat product, and moderate-to-severe HS
Carrieri et al., 2014, France [86]	Prospective, median FU 3.3 years	Coffee, chocolate	990 HIV/HCV coinfected patients (ANRS CO13 HEPAVIH cohort)	M 70%, age 45 (42–48) years	91% on cART, 71% with viral suppression	N/A	- High coffee (>3 cups/day) and chocolate intake were associated with lower transaminases levels
Carrieri et al., 2017, France [87]	Prospective, median FU 5 years	Coffee	1028 HIV/HCV coinfected patients (ANRS CO13 HEPAVIH cohort)	M 70%, age 49 (46–52) years	94.5% on ART, 82.5% with viral suppression	Liver fibrosis: FIB-4	- High coffee intake (>3 cups/day) was associated with a 50% reduction in all-cause mortality risk - High coffee intake was associated with lower prevalence of advanced liver fibrosis
Carrieri et al., 2018, France [88]	Cross-sectional	Coffee	918 HIV/HCV coinfected patients (ANRS CO13 HEPAVIH cohort)	N/A	N/A	HS: N/A Liver fibrosis: LSM by TE and FIB-4	- Higher coffee intake was associated with lower liver fibrosis, with an inverse dose–response relationship - No association between coffee intake and HS
Yaya et al., 2018, France [89]	Cross-sectional	Coffee	1019 HIV/HCV coinfected patients (ANRS CO13 HEPAVIH cohort)	M 70%, age 47.8 (±6.4) years	95% on cART	Liver fibrosis: FIB-4	- High coffee intake (>3 cups/day) was associated with lower odds of advanced liver fibrosis - High coffee intake mitigated harmful effects of alcohol consumption on liver fibrosis - Among patients with low-risk alcohol consumption, high coffee intake was associated with lower prevalence of advanced liver fibrosis compared to low coffee intake

**Legend**: Continuous variables are expressed as mean + standard deviation or median (interquartile range or range) and categorical variables are presented as percentages. Abbreviations: BMI, body mass index; HIV, human immunodeficiency virus; cART, combined antiretroviral therapy; NAFLD, non-alcoholic fatty liver disease; NASH, non-alcoholic steatohepatitis; PWH, people with HIV; M, males; F, females; FAs, fatty acids; FU, follow-up; HS, hepatic steatosis; CAP, Controlled Attenuation Parameter; N/A, not assessed; LSM, liver stiffness measurement; TE, transient elastography; and FIB-4, Fibrosis-4.

**Table 2:** Studies evaluating the role of micronutrients in hepatic steatosis and/or liver fibrosis in people with HIV.

Author, Year, Country	Study Design	Nutrients of Interest	Study Cohort	Patients’ Characteristics (Sex, Age, BMI)	HIV Features (Duration of HIV Infection, cART, Viral Suppression)	Diagnostic Method for NAFLD/ NASH/Liver Fibrosis	Main Findings
Sebastiani et al., 2020, Canada [105]	Single-center, open-label, single-arm, clinical trial, FU 4 years	Vitamin E	27 PWH with NASH	M 81%, age 54 (51–59) years, BMI 28 (25–32) kg/m^2^	Duration HIV 23 (15–29) years, 100% on cART, 100% with viral suppression	- NASH: CAP + cytokeratin 18 (100% of the cohort); liver biopsy (15% of the cohort) - Liver fibrosis: LSM by TE	- 24 months of vitamin E treatment improved liver inflammation, HS, and hepatocyte apoptosis - Good safety profile of vitamin E (800 IU/day for 24 months) - No improvement in BMI or liver fibrosis with vitamin E treatment
Guzman-Fulgencio et al., 2013, Spain [106]	Cross-sectional	Vitamin D	174 HIV/HCV-coinfected patients	M 75%, age 40.8 (37.3; 44.6) years,	86% on cART, 71% with viral suppression	Liver fibrosis: liver biopsy	- High prevalence of vitamin D insufficiency and deficiency (75.3% and 15.5%, respectively) - Vitamin D deficiency was independently associated with liver fibrosis severity
Terrier et al., 2011, France [107]	Cross-sectional	Vitamin D	189 HIV/HCV-coinfected patients	M 77%, Age 39.5 (±4.8) years, BMI 22.7 (±3.2) kg/m^2^	Duration HIV 12 (0.5–18.5) years, 82% on cART, 70% with <400 HIV-RNA copies	Liver fibrosis: liver biopsy	- High prevalence of vitamin D insufficiency and deficiency (63% and 23%, respectively) - Vitamin D deficiency was independently associated with liver fibrosis severity
Milazzo et al., 2011, Italy [108]	Cross-sectional	Vitamin D	237 patients - 144 HIV monoinfected (*HIV*) - 93 HIV/HCV-coinfected (*HIV*/*HCV*)	- *HIV*: M 65%, age 44 (39–50) years, BMI 23.8 (21.5–26.2) kg/m^2^ - *HIV/HCV*: M 74%, age 45 (43–48) years, BMI 23.27 (20.7–25.27) kg/m^2^	- *HIV*: Duration HIV 11.5 (6–15) years - *HIV/HCV*: Duration HIV 18 (13–22) years	Liver fibrosis: FIB-4, liver biopsy (20% of the cohort)	- Vitamin D deficiency was low in *HIV* and *HIV/HCV* (7.46% and 4.3%, respectively) - Vitamin D insufficiency was high in *HIV* and *HIV/HCV* (63.8% and 59%, respectively) - In *HIV/HCV,* vitamin D deficiency was independently associated with advanced liver fibrosis - Vitamin D deficiency was not associated with HCV coinfection or cART
Mandorfer et al., 2015, [109]	Cross-sectional	Vitamin D	86 HIV/HCV-coinfected patients	M 71%, age 38.7 (+9.6) years, BMI 23.2 (+4) kg/m^2^	71% on cART	Liver fibrosis: liver biopsy	Vitamin D deficiency was independently associated with liver fibrosis progression and higher portal pressure
El-Maouche et al., 2013 U.S. [110]	Cross-sectional	Vitamin D	116 HIV/HCV-coinfected patients	M 63%, age 49.9 (46.5–53.3) years	64% on cART, 79% with <400 HIV-RNA copies	Liver fibrosis: liver biopsy	- High prevalence of vitamin D deficiency (41%) - Vitamin D deficiency was not associated with liver fibrosis severity or low bone mineral density
Milic et al., 2020, Italy [111]	Cross-sectional	Vitamin D	707 PWH	M 76%, age 53.5 (±8.2) years, BMI 24.6 (±4.2) kg/m^2^	100% on cART, 99% with viral suppression	- HS: CAP - Liver fibrosis: LSM by TE	Vitamin D deficiency was associated with NAFLD with liver fibrosis
Nordmann et al., 2018, France [112]	Cross-sectional	Cannabinoids	838 HIV/HCV-coinfected patients (ANRS CO13 HEPAVIH cohort)	M 70%, age 44.9 (44.5–45.4) years	92% on cART, 84% with viral suppression	HS: abdominal ultrasound	Daily cannabis use was independently associated with lower prevalence of HS
Barré et al., 2021, France [113]	Prospective, FU 5 years	Cannabinoids	997 HIV/HCV-coinfected patients (ANRS CO13 HEPAVIH cohort)	N/A	N/A	HS: fatty liver index	Regular cannabis use was associated with a 55% lower risk of elevated fatty liver index
Fuster et al., 2021, Russia [114]	Cross-sectional	Cannabinoids	248 PWH with high prevalence of alcohol abuse (93%) and HCV coinfection (88%)	M 73%, age 33 (30–37) years, BMI 22.5 (20.8–24.5) kg/m^2^	N/A	Liver fibrosis: FIB-4, APRI, LSM by TE	Cannabis use was not associated with advanced liver fibrosis
Kelly et al., 2016, USA [115]	Prospective, FU 11 years	Cannabinoids	575 HIV/HCV-coinfected patients	*Entry visit*: F 100%, age 40 (±6) years, BMI 26 (±6) kg/m^2^*Last visit*: F 100%, age 51 (±7) years, BMI 26 (±7) kg/m^2^	*Entry visit*: 6% on cART, 7% with viral suppression *Last visit*: 63% on cART, 33% with viral suppression	Liver fibrosis: FIB-4, APRI	Cannabis use was not associated with progression to significant liver fibrosis
Brunet et al., 2014, Canada [116]	Prospective, FU 2.7 years	Cannabinoids	690 HIV/HCV-coinfected patients	M 73%, age 43.9 (38.4–49.2) years, BMI 24.0 (21.2–26.8) kg/m^2^	Duration HIV 18.0 (10.4–24.5) years, 54% with viral suppression	Liver fibrosis and cirrhosis: APRI	Cannabis use was not associated with liver fibrosis progression or cirrhosis

**Legend:** Continuous variables are expressed as mean + standard deviation or median (interquartile range or range) and categorical variables are presented as percentages. Abbreviations: BMI, body mass index; HIV, human immunodeficiency virus; cART, combined antiretroviral therapy; NAFLD, non-alcoholic fatty liver disease; NASH, nonalcoholic steatohepatitis; FU, follow-up; PWH, people with HIV; M, males; CAP, Controlled Attenuation Parameter; HS, hepatic steatosis; ALT, alanine transaminase; FIB-4, Fibrosis-4; LSM, liver stiffness measurement; TE, transient elastography; and APRI, AST to Platelet Ratio Index.

**Table 3:** Studies evaluating the role of lifestyle intervention in weight loss in hepatic steatosis in people with HIV.

Author, Year, Country	Study Design	Lifestyle Intervention	Study Cohort	Patients’ Characteristics (Sex, Age, BMI)	HIV Features (Duration of HIV Infection, cART, Viral Suppression)	Diagnostic Method for NAFLD/ NASH	Main Findings
Sebastiani et al., 2020, Canada [105]	24-week single-center, open-label, single-arm, clinical trial; FU 4 years	At baseline standardized dietary and physical activity recommendations plus a nutritional consultation	27 PWH with NASH receiving vitamin E	M 81%, age 54 (51–59) years, BMI 28 (25–32) kg/m^2^	Duration HIV 23 (15–29) years, 100% on cART, 100% with viral suppression	NASH: CAP + cytokeratin 18 (100% of the cohort); liver biopsy (15% of the cohort)	Standard lifestyle recommendations with nutritional consultations were not associated to BMI changes, after 48 months of follow-up
Stanley et al., 2019, USA [178]	48-week, randomized, double-blind, multicenter trial	Nutritional counselling from clinical research nutritionists at baseline, 6 months, and 12 months	61 PWH with NAFLD: - Tesamorelin (n = 30) - *Placebo* (n = 30) *	- *Tesamorelin*: M 77%, age 52 (±8) years, BMI 30 (±6) kg/m^2^ - *Placebo*: M 80%, age 54 (±7), BMI 33 (±6) kg/m^2^	- *Tesamorelin*: duration HIV 16 (±9), 100% on cART, 100% with viral suppression - *Placebo*: duration HIV 18 (±8), 100% on cART, 100% with viral suppression	NAFLD by HFF ≥ 5% with MRS	Nutritional counselling was not associated with significant weight loss, waist circumference reduction, changes in daily caloric, macronutrient, and alcohol intake or hours of physical activity, after 48 months of follow-up
Policarpo et al., 2021, Portugal [180]	Single-center, randomized control trial	Detailed tailored nutritional plan based on MD with a caloric deficit of 500 kcal driven by the nutritionist, plus exercise training counselling	55 PWH with NAFLD - Standard of care (n = 28) - Lifestyle intervention (n = 27)	- *Standard of Care*: M 71%, age 55.9 (±11.7) years, BMI 28.1 (±4.0) kg/m^2^ - *Lifestyle intervention*: M 74%, age 52.6 (±9.9) years, BMI 27.7 (±4.9) kg/m^2^	- *Standard of Care*: Duration HIV 17.2 (±7.1) years - *Lifestyle intervention*: Duration HIV 18.4 (±8.1) years	NAFLD by abdominal ultrasound	- Lifestyle intervention was significantly associated with weight loss compared to standard of care (−1.5 kg vs. +0.65 kg, *p* < 0.001), after 5 months of follow-up - Lifestyle intervention with telemedicine follow-up mitigated the negative effects of lockdown on weight gain
Yarasheski et al., 2011, USA [185]	Single-center, randomized control trial	Exercise training program based on 1.5–2 h/day, 3 days/week, of supervised, progressive, combined aerobic conditioning and resistance training driven by a certified exercise trainer	39 PWH with insulin resistance and central adiposity: - Pioglitazone (n = 20) - Pioglitazone + exercise training (n = 19)	- *Pioglitazone*: M 85%, age 44 (±2), BMI 29.1 (±1.2) kg/m^2^ - *Pioglitazone + exercise training*: M 89%, age 46 (±2), BMI 28.3 (±1.2) kg/m^2^	- *Pioglitazone*: duration HIV 11 (±1), 100% with viral suppression - *Pioglitazone + exercise training*: duration HIV 13 (±1), 95% with viral suppression	HS by ^1^H-MRS	- Structured exercise training improved the beneficial effects of pioglitazone on peripheral insulin sensitivity - Pioglitazone plus exercise reduced liver lipid content more than pioglitazone alone (−2.6 ± 1.4% vs. −1.5 ± 1.3, *p* = 0.6)

* One patient withdrew consent **Abbreviations**: HIV, human immunodeficiency virus; BMI, body mass index; NAFLD, non-alcoholic fatty liver disease; NASH, non-alcoholic steatohepatitis; FU, follow-up; PWH, people with HIV; CAP, Controlled Attenuation Parameter; HFF, hepatic fat fraction; MRS, magnetic resonance spectroscopy; HS, hepatic steatosis; ^1^H-MRS, proton magnetic resonance spectroscopy; and MD, Mediterranean diet.

**Table 4:** Clinical trials evaluating the pharmacological treatment of NAFLD/NASH in people with HIV.

Author, Year, Country	Study Design	Treatment	Sample Size	Diagnostic Method for NAFLD/NASH/Liver Fibrosis	Main Findings
Sebastiani et al., 2020, Canada [105]	24-week, single-center, open-label, single-arm trial	Vitamin E	27 PWH with NASH	- NASH by CAP ≥ 248 dB/m and cytokeratin 18 > 130.5 U/L - Liver fibrosis by LSM at TE	24-week vitamin E treatment decreased liver inflammation (with ALT −27 units/L), steatosis (with CAP −22 dB/m), and hepatocyte apoptosis (with cytokeratin 18–123 units/L)
Matthews et al., 2015, U.S. [186]	48-week, randomized, double-blind, placebo-controlled trial	Pioglitazone	13 HIV/HCV-coinfected patients with NAFLD: - Pioglitazone (n = 6) - Placebo (n = 7)	NAFLD by HFF ≥ 5% with MRS	48-week pioglitazone treatment significantly decreased hepatic fat from baseline (15.1% ± 7.0%) to week 48 (7.6% ± 3.9%), with a mean difference of −7.4% (*p* = 0.02), whereas placebo administration did not change hepatic fat content
Kamolvisit et al., 2021, Thailand [187]	48-week, randomized, placebo-controlled trial	Pioglitazone	98 PWH with prediabetes and MAFLD: - Pioglitazone (n = 49) - Placebo (n = 49)	- MAFLD by CAP ≥ 238 dB/m or by abdominal ultrasound - Liver fibrosis by LSM at TE	48-week pioglitazone treatment decreased HS and liver fibrosis, with a significantly higher change in CAP and LSM compared to placebo (−23.5 dB/m vs. 10.2 dB/m, *p* < 0.001 and −0.184 kPa vs. 0.554 kPa, *p* = 0.0016, respectively)
Ajmera et al., 2019, U.S. [188]	12-week, double-blind, randomized, investigator-initiated, placebo-controlled trial	Aramchol	50 PWH with NAFLD: - Aramchol (n = 25) - Placebo (n = 25)	- NAFLD by MRI- PDFF ≥ 5% - Liver fibrosis by MRE and VCTE	12-week Aramchol treatment did not reduce HS and liver fibrosis or change body fat and muscle composition
Stanley et al., 2019, U.S. [178]	48-week, randomized, double-blind, multicenter trial	Tesamorelin	61 PWH with NAFLD: - Tesamorelin (n = 30) - Placebo (n = 30) *	- NAFLD by HFF > 5% with MRS - Liver fibrosis and NASH by liver biopsy	48-week tesamorelin treatment reduced hepatic fat content by 37% and prevented progression of liver fibrosis, with a significantly lower rate of liver fibrosis progression in the tesamorelin group compared to placebo (10.5% vs. 37.5%, *p* = 0.04)
MASH study, 2020, UK [189]	48-week, multicenter, open-label, single-arm trial	Maraviroc	13 PWH with NASH	NASH and liver fibrosis by liver biopsy	48-week maraviroc treatment did not change HS, liver fibrosis, or NASH features

* 1 patient withdrew consent. **Abbreviations**: HIV, human immunodeficiency virus; NAFLD, non-alcoholic fatty liver disease; NASH, non-alcoholic steatohepatitis; PWH, people with HIV; CAP, Controlled Attenuation Parameter; LSM, liver stiffness measurements; TE, transient elastography; ALT, alanine transaminase; U.S., United States; HS, hepatic steatosis; HFF, hepatic fat fraction; MRS, magnetic resonance spectroscopy; MAFLD, metabolic (dysfunction)-associated fatty liver disease; MRI-PDFF, magnetic resonance imaging-proton density fat fraction; MRE, magnetic resonance elastography; VCTE, vibration-controlled transient elastography; MASH, maraviroc add-on for steatohepatitis in HIV-infected patients; and UK, United Kingdom.

## Data Availability

Not applicable.

## Supplementary Materials

The following supporting information can be downloaded at: https://www.mdpi.com/article/10.3390/nu15081990/s1, Figure S1: The role of food insecurity on health outcomes.
