# Supplementary material for: Nutritional and Lifestyle Therapy for NAFLD in People with HIV
Source: Nutrients. 2023 Apr 20;15(8):1990. doi: 10.3390/nu15081990 (PMC10140991; doi:10.3390/nu15081990)
Supplement: Supplementary file 1 [file nutrients-15-01990-s001.zip › nutrients-2339183-supplementary.pdf]

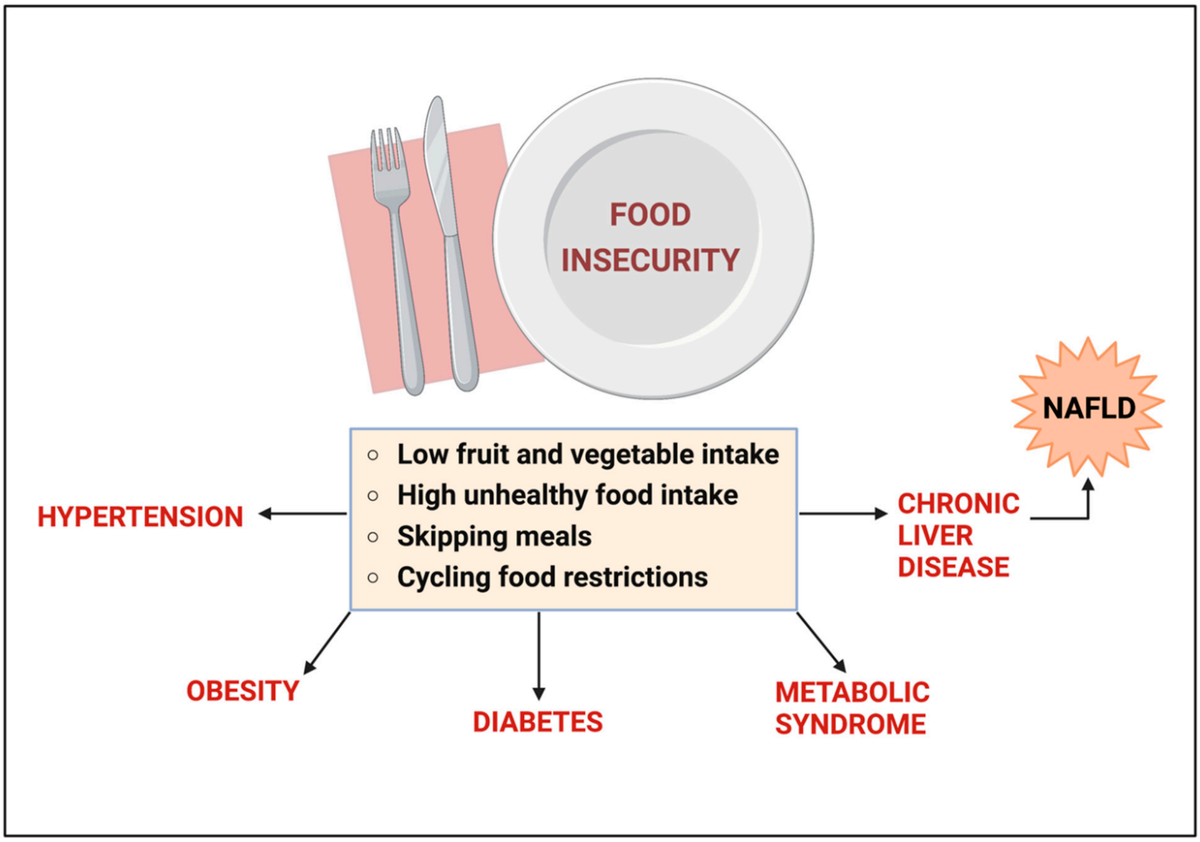

Figure S1. The role of food insecurity on health outcomes. Food insecurity, which is the lack of consistent access to enough food for every person in a household to live an active and healthy life, is associated with poor health outcomes. In fact, food insecurity leads to lower fruits and vegetables intake, skipping meals and cycling food restrictions, along with higher consumption of inexpensive junk foods with poor nutritional values. Therefore, it is associated to an augmented cardio-metabolic risk, with high prevalence of hypertension, obesity, diabetes, metabolic syndrome, and chronic liver disease. Food insecurity seems also to be associated with increased odds for NAFLD in PWH. *Abbreviations*: NAFLD Non-Alcoholic Fatty Liver Disease, PWH People With HIV.
